# Simple diameters, accurate stages:a practical approach to HD-ISS staging

**DOI:** 10.1007/s00415-025-13409-1

**Published:** 2025-11-14

**Authors:** Beatrice Heim, Florian Krismer, Greta Hemicker, Clancy Cerejo, Katarina Schwarzova, Samuel Labrecque, Federico Carbone, Marina Peball, Christoph Scherfler, Philipp Mahlknecht, Astrid E. Grams, Elke R. Gizewski, Atbin Djamshidian, Klaus Seppi

**Affiliations:** 1 Department of Neurology, Medical University of Innsbruck, Anichstrasse 35, 6020 Innsbruck, Austria; 2 Department of Radiology, Medical University of Innsbruck, Innsbruck, Austria; 3 Neuroimaging Research Core Facility, Medical University of Innsbruck, Innsbruck, Austria

**Keywords:** Huntington’s disease, HD-ISS, MRI, Biomarkers

## Abstract

**Background:**

Huntington’s disease (HD) is a neurodegenerative disorder caused by CAG repeat expansions. The Huntington’s Disease Integrated Staging System (HD-ISS) classifies disease stages (0–3) using clinical, biological, and imaging assessments, with caudate and putamen volumes serving as key early biomarkers. Conventional automated volumetric methods typically require specialized software and experienced operators, limiting broader accessibility. Simple linear measurements, such as the bicaudate ratio (BCR) and bifrontal-to-bicaudate ratio (FCR), may provide a practical alternative for assessing striatal atrophy.

**Methods:**

Reference caudate and putamen volumes were measured using automated segmentation (FreeSurfer). Linear measurements (frontal horn width, intercaudate distance, inner table width) were used to calculate BCR and FCR in a training cohort of 44 HD mutation carriers. Regression models predicting caudate and putamen volumes based on FCR were developed and validated in an independent test cohort of 11 HD mutation carriers. Interrater reliability was assessed via intraclass correlation coefficients (ICC).

**Results:**

Interrater reliability was excellent. Among linear measurements, FCR had the strongest correlation with caudate (r = 0.866; R^2^ = 0.749) and putamen volumes (r = 0.897; R^2^ = 0.805). Predictive equations derived from FCR-based regression models accurately estimated volumes, resulting in identical HD-ISS staging classifications compared to actual volumetric measurements for all test cohort participants.

**Conclusion:**

FCR-based linear estimation of caudate and putamen volumes provides accurate and reliable HD-ISS staging. This simplified method offers a practical and time-efficient alternative to conventional volumetry, potentially facilitating wider application of HD-ISS staging in clinical practice and non-specialist research settings.

## Introduction

Huntington’s disease (HD) is a progressive neurodegenerative disorder caused by an expanded CAG trinucleotide repeat in the HTT gene on chromosome 4, leading to the production of mutant huntingtin protein (mHtt). While clinical diagnosis traditionally relies on the emergence of extrapyramidal motor symptoms, non-motor manifestations—such as cognitive decline and behavioral disturbances—often precede motor signs by years or even decades [1].​

To better capture the full disease continuum, the Huntington’s Disease Integrated Staging System (HD-ISS) was introduced as a standardized framework for clinical research [2]. It integrates biological, clinical, and functional markers to classify HD mutation carriers into stages 0 through 3, with higher stages indicating greater clinical impairment. Notably, the HD-ISS incorporates structural magnetic resonance imaging (MRI) biomarkers, such as caudate and putamen volumes, for early disease staging.​

However, obtaining these volumetric measures can be challenging in routine clinical practice. Conventional approaches, such as manual or semi-automated segmentation of regions of interest (ROIs), are time-intensive and operator-dependent [3]. Fully automated methods using software such as FreeSurfer offer greater consistency [4, 5], but they require high-quality MRI data and computing infrastructure, limiting their use to expert centers.

Historically, simple linear brain measurements such as the bicaudate ratio (BCR) and the bifrontal-to-bicaudate ratio (FCR) have been used as surrogate markers of striatal atrophy in HD, especially prior to the widespread availability of automated volumetric imaging tools.

The BCR, calculated as the minimum intercaudate distance divided by the inner table width of the skull, was one of the earliest quantitative imaging markers associated with disease progression in HD. Earlier studies demonstrated that increased BCR values correlate with striatal atrophy and clinical severity in HD patients, making it a useful tool for distinguishing affected individuals from controls and for monitoring disease progression over time [3, 6].​ Similarly, the FCR, which incorporates both frontal horn width and bicaudate distance, was introduced to enhance sensitivity to early frontostriatal changes. These linear measures were particularly valuable in clinical and research settings with limited access to high-resolution imaging or post-processing capabilities. Although they were gradually replaced by more precise volumetric methods, recent interest has re-emerged in these simpler techniques as potential low-cost, scalable markers suitable for widespread clinical use—especially considering staging systems like HD-ISS that depend on structural brain measures.​

The implementation of a structural MRI–based staging paradigm for HD offers notable advantages for both clinical management and research. Adaptation of the HD-ISS framework for rapid, reproducible use outside specialized imaging centers enables sensitive detection of disease-specific neuroanatomical alterations, including prodromal changes, supports standardized and longitudinal biomarker acquisition, and strengthens the evidentiary basis for therapeutic stratification, patient counseling, and clinical trial enrollment. By providing a robust and clinically feasible staging methodology, this approach has the potential to enhance patient outcomes and accelerate translational advances in HD.

​The objective of this study was to assess whether simple 1D MRI measures can serve as reliable proxies for caudate and putamen volumes, thereby enabling broader clinical and research application of HD-ISS staging. Rather than replacing volumetric techniques, we propose a low-cost, workflow-compatible alternative for settings where automated volumetry is constrained by infrastructure, time, or licensing requirements. Standardized linear measures of striatal atrophy may provide a practical solution to support HD-ISS–aligned staging, longitudinal monitoring, and patient recruitment, particularly outside specialized imaging centers.

## Methods

### Study population

In this retrospective, cross-sectional study, we evaluated 55 genetically confirmed HD mutation carriers. Eligible participants were selected from patients followed at our outpatient facilities between 2010 and 2024. The main inclusion criteria included availability of a high-field (3.0 Tesla) MRI examination performed at our institution, specifically including a three-dimensional magnetization-prepared rapid gradient-echo (3D-MPRAGE) T1-weighted sequence. Given the clinical relevance of MRI volumetry in distinguishing HD-ISS stage 0 from stage 1, we deliberately restricted a test cohort to premanifest HD mutation carriers. To ensure random allocation, premanifest participants were distributed to training and test cohorts in a 1:4 ratio (approximately 75% to the training set and 25% to the test set) using Excel’s RANDBETWEEN(1, 4) function. An output of ‘1’ resulted in assignment to the test set, while all other outputs led to assignment to the training set. This approach allowed robust imaging biomarker evaluation in the premanifest test set and preserved a large manifest training set for stable model development.

### Clinical assessments

Clinical assessments, including the Unified Huntington’s Disease Rating Scale Total Functional Capacity (UHDRS-TFC) and Total Motor Score (UHDRS-TMS), were performed at the time of the MRI examination or within a maximum interval of ± 3 months from the MRI date, ensuring that clinical staging closely corresponded to the structural imaging data used in analyses.

Participants were classified as *manifest HD* if they had a UHDRS-TMS > 5 and a diagnostic confidence level (DCL) of 4, and as *premanifest HD* if they had a UHDRS-TMS < 5 and a DCL < 4. Participants were further separated into clinical disease stages according to their functional capacities (measured by UHDRS-TFC) [7]: stage 1: UHDRS-TFC 13–11; stage 2: UHDRS-TFC 10–7; stage 3: UHDRS-TFC 6–3; stage 4: UHDRS-TFC 2–1; stage 5: UHDRS-TFC 0.

### Magnetic resonance imaging protocol and imaging analysis

All participants underwent high-resolution structural MRI using clinical 3.0 Tesla scanners (Verio or Skyra, Siemens Healthineers, Erlangen, Germany). While efforts were made to ensure consistent imaging quality, retrospective data acquisition over a 14-year period resulted in some variability in sequence parameters and hardware configurations, including the use of different head coils and potential differences across more than five protocol variants. Detailed coil type could not be reliably extracted from DICOM metadata due to anonymization and PACS export variability.

All scans included a T1-weighted 3D magnetization-prepared rapid gradient echo (MPRAGE) sequence with the following parameters: TR = 1800 ms; TE = 2.18 ms; inversion time (TI) = 900 ms; slice thickness = 1.2 mm; matrix = 256 × 204; flip angle = 9°; field of view = 220 × 165 mm. Minor deviations in parameter settings across scanners may introduce limited variability in volumetric estimates (~ 5%), as previously reported. However, all included scans passed visual quality control and met the minimum criteria for structural brain analysis.

The different 1D measurements including the frontal horn width (FHW), the intercaudate distance (ICD) and the inner table width (ITW) to determine BCR and FCR were measured according to methods described previously [4].

All linear measurements were performed on our institutional PACS workstation (IMPAX, Agfa HealthCare) using the built-in distance measurement tools. The measurement procedure is not dependent on this specific PACS; identical results can be obtained on any clinical imaging platform equipped with basic caliper functionality. IMPAX was chosen in this study as it reflects the standard tools available in routine clinical practice and ensures that the method can be readily implemented without specialized research software or additional infrastructure. For obtaining the ratios, a standardized protocol was used: all measurements were performed on AC–PC–aligned 3D T1-weighted images. In the axial plane at the level of the foramen of Monro, the slice showing the smallest distance between the caudate heads (intercaudate distance, ICD) was identified and confirmed in orthogonal views. On this slice, the minimum ICD and the inner table width (ITW) of the skull were measured. The frontal horn width (FHW), defined as the distance between the lateral margins of the frontal horns, was measured on the axial slice corresponding to the bifrontal line. From these measurements, the bicaudate ratio (BCR; ICD/ITW) and the bifrontal-to-bicaudate ratio (FCR; FHW/ICD) were calculated. All raters underwent brief training using reference examples prior to data collection. Measurement time per case was approximately 1–2 min after AC–PC alignment. The different 1D measurements in the training set were performed by a senior rater (KS: n = 16; or BH: n = 28) and two junior raters (CC and GH), and in the test set by the two senior rater (KS and BH).

Putaminal and caudate volumes as well as the intracranial volumes (ICV) were measured with FreeSurfer 6.0.1, which is a well-established neuroimaging analysis suite for cortical reconstruction and volumetric segmentation. The technical details of these procedures are described in prior publications [8–18].

Briefly, this processing includes motion correction and averaging [8] of multiple volumetric T1 weighted images (when more than one is available), removal of non-brain tissue using a hybrid watershed/surface deformation procedure [13], automated Talairach transformation, segmentation of the subcortical white matter and deep gray matter volumetric structures (including hippocampus, amygdala, caudate, putamen, ventricles) [17, 18], intensity normalization [19], tessellation of the gray matter white matter boundary, automated topology correction [10, 20], and surface deformation following intensity gradients to optimally place the gray/white and gray/cerebrospinal fluid borders at the location where the greatest shift in intensity defines the transition to the other tissue class [11, 14, 21]. Once the cortical models are complete, a number of deformable procedures can be performed for further data processing and analysis including surface inflation [12], registration to a spherical atlas which is based on individual cortical folding patterns to match cortical geometry across subjects [22], parcellation of the cerebral cortex into units with respect to gyral and sulcal structure [23, 24], and creation of a variety of surface based data including maps of curvature and sulcal depth. This method uses both intensity and continuity information from the entire three-dimensional MR volume in segmentation and deformation procedures to produce representations of cortical thickness, calculated as the closest distance from the gray/white boundary to the gray/CSF boundary at each vertex on the tessellated surface [14]. The maps are created using spatial intensity gradients across tissue classes and are therefore not simply reliant on absolute signal intensity. FreeSurfer morphometric procedures have been demonstrated to show good test–retest reliability across scanner manufacturers and across field strengths [9, 16]. Caudate and putaminal volumes are reported as a proportion of the estimated total intracranial volume (ICV).

### Statistics

Data analysis was performed using SPSS 29. Gaussian distribution was confirmed by visual interpretation of the Q–Q (quantile–quantile) plots and the Kolmogorov–Smirnov test. Demographic data are presented as frequencies, means ± standard deviations, or median (interquartile range) according to data distribution. Group differences for demographic variables, clinical variables, and imaging measures (putamen and caudate volumina as a proportion of the ICV as well as BCR and FCR) were assessed using parametric tests (analysis of variance [ANOVA] or unpaired t-tests) for continuous and normally distributed variables, nonparametric tests (Kruskal–Wallis one-way ANOVA by ranks or Mann–Whitney U test) for non-normal continuous variables, and Pearson's chi-square tests for categorical variables across different disease stages of all HD mutation carriers.

The interrater variability in the test cohort was assessed by comparing the different 1D measurements between a senior rater (KS or BH) and two less experienced raters (CC and GH). The interrater reliability was calculated using interclass correlation coefficients (ICCs) using a two‐way mixed‐effects analysis of variance model, with values interpreted as follows [25]: ICC values < 0.50 were considered poor, values between 0.50 and 0.75 were considered moderate, values between 0.75 and 0.90 were considered good, and values > 0.90 were considered excellent.

To exclude any sex-differences for the ratios derived from the 1D measurements, we performed a univariate ANOVA with FCR and BCR as dependent variables and sex as the independent variable, correcting for staging according to the Shoulson–Fahn system (premanifest versus stage 1 versus stage ≥ 2).

Pearson correlation analyses were performed to assess the relationships between caudate and putamen volumes (as a proportion of the ICV) and the manually measured ratios BCR and FCR in the training cohort. The ratio that showed the strongest correlation with caudate and putamen volumes was selected for further analysis using linear regression. Linear regression analysis was employed to quantify the relationship between caudate and putamen volumes (dependent variables) and the manually measured ratio (independent variable) in the training cohort. The regression coefficients were then used to develop predictive equations for estimating caudate and putamen volumes as a proportion of the ICV based on the independent variables according to the formula y = α + β * x.

Paired t-tests were conducted to perform within-subject comparisons of caudate and putamen volumes in the training set, as well as the predicted caudate and putamen volumes derived from linear regression analysis in both the training and the test set.

The test set was used for classification according to the HD-ISS staging system. Caudate and putamen volumes (as a proportion of the ICV) in the test set were entered into the HD-ISS calculator (https://enroll-hd.org/calc/html_basic.htm). Additionally, predicted caudate and putamen volumes were calculated using the formulas derived from the linear regression analysis in the training set. These predicted volumes were then entered into the HD-ISS calculator to determine whether the HD-ISS stage differed when calculated using actual versus predicted caudate and putamen volumes. This procedure was additionally performed using measurements from the second senior rater (BH).

## Results

### Clinical and demographic data

Demographic, clinical and imaging data of the whole study cohort are summarized in Table 1.

**Table 1:** Demographic characteristics of all participants by clinical disease stage

	preHD	Clinical stage 1	Clinical stage 2	p-value
Number (n)	15	20	20	
Male/Female	4/11	13/7	10/10	0.087^1^
Age (years) (± SD)	37.20 ± 7.36	46.35 ± 12.01	50.00 ± 9.31	< 0.001^2^
UHDRS-TMS (± SD)	1.20 ± 1.70	17.50 ± 7.46	64.56 ± 13.08	< 0.001^3^
UHDRS-TFC (± SD)	13.00 ± 0.00	12.55 ± 0.83	5.56 ± 0.91	< 0.001^3^
CAG repeats (± SD)	43.93 ± 1.49	46.15 ± 4.23	47.10 ± 2.92	0.077^3^
CAP Score (± SD)	515.13 ± 94.86	708.45 ± 71.32	830.25 ± 117.27	< 0.001^2^
PIN_HD_ (± SD)	0.08 ± 0.74	2.41 ± 1.14	4.53 ± 1.74	< 0.001^2^
SDMT (± SD)	49.14 ± 14.15	36.82 ± 16.02	21.14 ± 8.85	< 0.001^3^
Educational years (± SD)	14.01 ± 2.51	13.22 ± 2.80	11.69 ± 2.05	< 0.001^2^
FCR (±SD)	2.28±0.49	1.76±0.28	1.60±0.19	<0.001^2^
BCR (±SD)	0.13±0.03	0.18±0.03	0.20±0.03	<0.001^2^
Adjusted caudate volume^*^	3.72±0.74	2.61±0.52	2.40±0.68	<0.001^2^
Adjusted putaminal volume^*^	5.26±1.06	3.85±0.53	3.51±0.56	<0.001^2^

^1^ chi-square test; ^2^ univariate ANOVA; ^3^ Kruskal–Wallis one-way ANOVA

^*^Adjusted caudate and putaminal volumes are reported as a proportion of the estimated total intracranial volume

**Staging:** clinical stage 1: TMS>5, TFC 13-11; clinical stage2: TMS>5, TFC<11.

**Abbreviations:** n Number of individuals; ANOVA analysis of variance; *BCR* bicaudate ratio; CAG– Cytosine-Adenine-Guanine trinucleotide repeat length in the HTT gene; FCR, bifrontal-to-bicaudate ratio (FCR); UHDRS-TMS – Unified Huntington’s Disease Rating Scale – Total Motor Score; UHDRS-TFC – Unified Huntington’s Disease Rating Scale – Total Functional Capacity; CAP Score (±SD) – CAG-Age Product Score; PINHD (±SD) – Prognostic Index Normalized for Huntington's Disease; SDMT (±SD) – Symbol Digit Modalities Test

We included 55 HD mutation carriers: 15 in the premanifest stage (one of whom was classified as HD-ISS stage 2 due to reduced SDMT) and 40 in the manifest stage, comprising 20 individuals in stage 1 and 20 in stage 2 or higher, according to the clinical classification [26]. As expected, TMS scores were lower and SDMT performance higher in stage 1 compared with more advanced stages.

### Training and test set composition

Of the 55 HD mutation carriers included in the study, 44 were assigned to the training set and 11 to the independent test set. The training cohort comprised mainly manifest patients (stage 1: n=20; stage ≥2: n=20), ensuring a sufficiently large dataset for stable model development. In contrast, the test set was deliberately enriched with premanifest carriers (n = 11) to enable independent evaluation of biomarker performance in an early disease subgroup. Random allocation of premanifest participants was performed in a 1:4 ratio using Excel’s RANDBETWEEN [1, 4] function. Linear MRI measurements in the training set were performed by two senior (KS, BH) and two junior raters (CC, GH), while in the test set only the senior raters (KS, BH) carried out the assessments.

### Interrater reliability of the 1D measurements

Both senior raters measured the 1D measurements in the 16 HD mutation carriers of the test cohort. The ICCs of all 1D measurements between the two senior raters were excellent (FHW: 0.974; ICD: 0.971; ITW: 0.930). There was excellent agreement (> 0.900) between all 1D measurements between the two junior raters and the senior rater except for FHW (0.850) as assessed by one junior rater.

### MRI data

Results of 1D measurements, BCR and FCR as well as putamen and caudate volumes (as a proportion of the ICV) are summarized in Table 1. Overall, it took 29.02 s (± 2.16) for the measurement of the FHW, 27.83 s (± 3.24) of the ICD and 32.57 s (± 4.83) of the ITW, respectively, as assessed in 10 mutation HD carriers. As expected, all putamen and caudate volumes as well as BCR and FCR were higher in the premanifest compared to the manifest HD mutation carriers (see Table 2). Univariate ANOVA, corrected for staging according to the Shoulson–Fahn system, revealed no significant sex differences for FCR (F = 0.110, p = 0.771) or BCR (F = 0.144, p = 0.740).

**Table 2:** Variables of the patients included in the training set (n = 11)

Age (years)	36	33	31	38	36	49	31	41	33	38	35
Sex	female	female	female	male	male	female	female	female	female	female	male
CAG repeats	45	45	44	47	42	44	45	42	45	42	44
UHDRS-TMS	0	0	0	2	0	4	4	1	0	0	2
UHDRS-TFC	13	13	13	13	13	13	13	13	13	13	13
UHDRS-IS	100	100	100	100	100	100	100	100	100	100	100
SDMT	56	58	49	44	87	51	47	31	33	49	52
Education level^#^	1	0	1	0	1	1	0	0	0	0	0
FCR	1.88	2.00	2.73	1.78	3.04	1.65	2.34	1.96	2.00	2.28	3.02
Volumes (× 10^–3^) by FreeSurfer*							4.14 4.99	3.34 4.01	3.18 4.43	3.65 4.40	4.18 6.09
Caudate	3.04	3.60	4.31	2.77	4.10	2.00					
Putamen	4.39	4.71	6.19	3.89	5.76	2.62					
Volumes predicted (× 10^–3^)											
Caudate	2.94	3.15	4.38	2.78	4.91	2.56	3.72	3.08	3.15	3.63	4.87
Putamen	4.03	4.29	5.86	3.82	6.53	3.55	5.02	4.21	4.29	4.90	6.48
Clinical stage	0	0	0	0	0	0	0	0	0	0	0
HD-ISS stage	1	1	0	1	0	1	1	1	1	1	0
HD-ISS stage according to predicted volumes	1	1	0	1	0	1	1	1	1	1	0

^#^ 0 = high school or less, 1 = more than high school

^*^ reported as a proportion of the estimated total intracranial volume (ICV)

Clinical stage: 0 (premanifest): TMS < 5; 1 (early stage): TMS > 5, TFC 13–11; 2 (middle stage): TMS > 5, TFC 7–10

*CAG* Cytosine-Adenine-Guanine trinucleotide repeat length in the HTT gene, *UHDRS-TMS* Unified Huntington’s Disease Rating Scale Total Motor Score, *UHDRS-TFC* Unified Huntington’s Disease Rating Scale Total Functional Capacity, *UHDRS-IS* Unified Huntington’s Disease Rating Scale Independence Scale, *SDMT* Symbol Digit Modalities Test, *FCR* Frontal-to-Caudate Ratio, *HD-ISS* Huntington’s Disease Integrated Staging System, *SD* Standard Deviation

### Predictive equations for estimating caudate and putamen voluminal

**Fig. 1:**
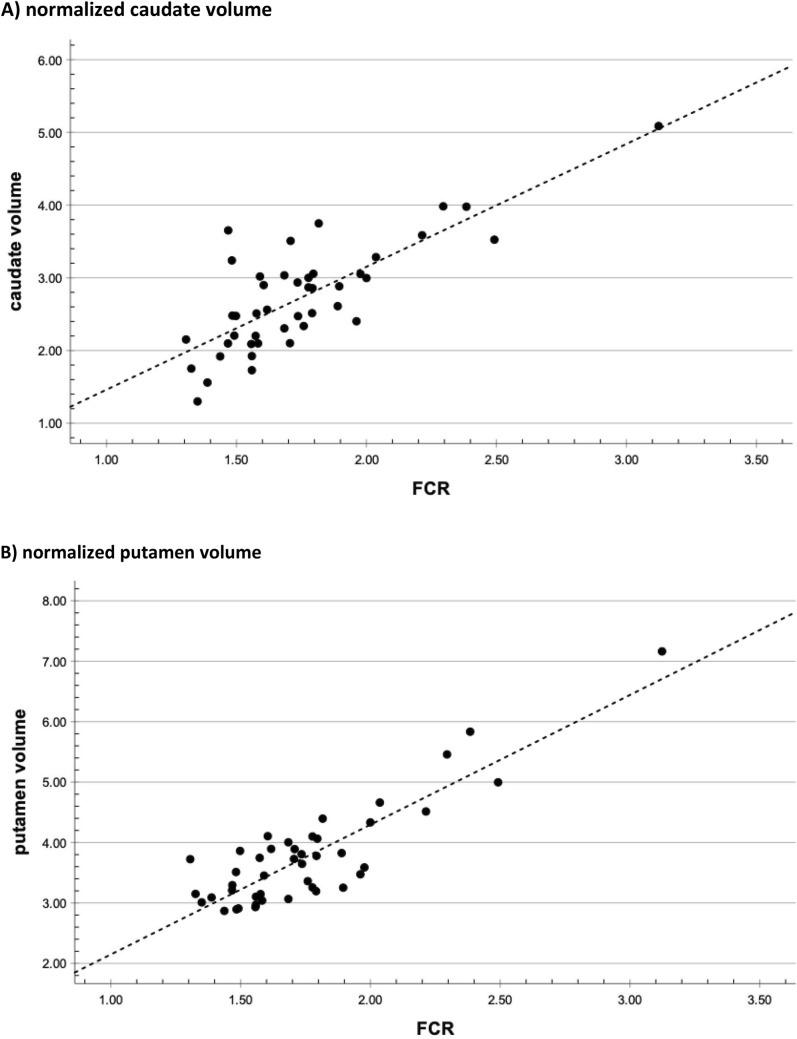
Validation of the regression formula: linear relationship between frontal caudate ratio (FCR) and normalized caudate and putamen volumes in the test cohort. **A** normalized caudate volume Normalized caudate volume (caudate volume / intracranial volume × 10^⁻3^) shows a strong linear correlation with the FCR (R^2^ = 0.616) in the independent test cohort. The regression line is defined by the equation: Caudate volume = ‘-0.229 + (1.690 × FCR), demonstrating the predictive validity of the model. **B** normalized putamen volume Normalized putamen volume (FreeSurfer-derived, scaled × 1000) shows a strong linear association with the FCR (R^2^ = 0.751). The regression line is defined by the equation: Putamen volume = 2.147 × FCR, demonstrating the model’s predictive strength

Pearson correlation coefficients (all p < 0.001) revealed that the FCR correlated better with caudate (r = 0.785) and putamen volumes (r = 0.867) than the BCR (r = 0.672 for caudate and 0.694 for putamen volumes). The FCR was therefore entered in the linear regression analysis to quantify its linear relationship between caudate (R^2^ = 0.616) and putamen (R^2^ = 0.751) volumes as dependent variables. The linear regression formula for predicting putamen and caudate volumes were ‘2.147 × FCR’ and ‘-0.229 + (1.690 × FCR)’, respectively. Within-subject comparisons of caudate and putamen volumes as well as the predicted caudate and putamen volumes from the FCR did not differ as shown in Fig. 1, neither in training nor the test set.

### Staging according to HD-ISS

**Fig. 2:**
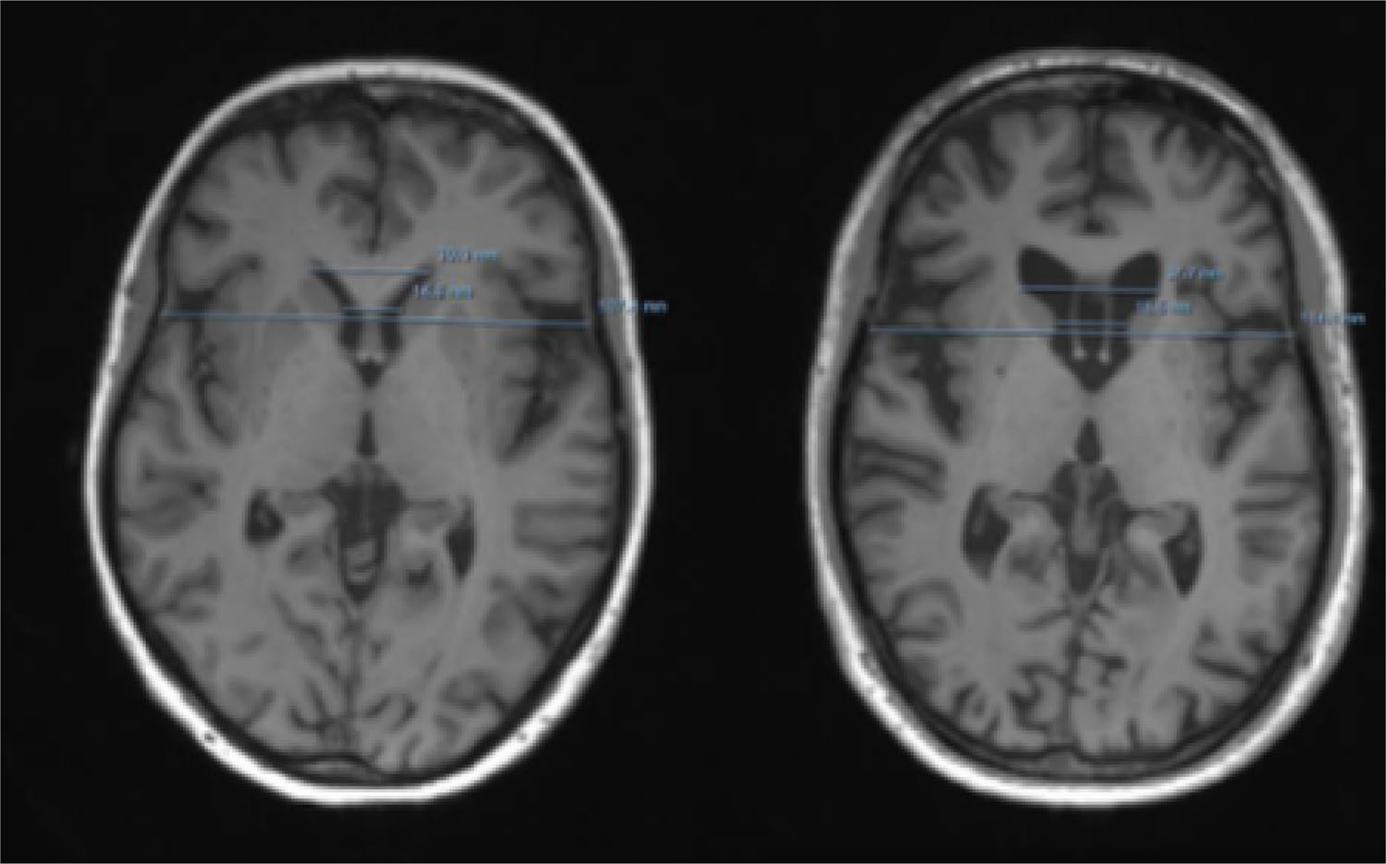
Example of the performed measurements in two different participants

Table 2 summarizes each of the 11 patients of the test set and the corresponding HD-ISS stage. The relevant clinical and imaging data including the caudate and putamen volumes and the predicted caudate and putamen volumes from the FCR are presented). Figure 2 shows an example of performed measurements in two different participants.

## Discussion

In this study, we demonstrate that a widely used [3, 4, 27], simple, linearly derived measurement—the bifrontal-to-bicaudate ratio (FCR)—can reliably estimate striatal volumes and serve as a practical surrogate for automated volumetric analysis of the caudate and putamen in HD. Among the linear parameters assessed, the FCR showed the strongest correlation with both caudate and putamen volumes and was thus selected for further analysis. The regression models based on FCR yielded robust results (R^2^ = 0.749 for caudate, R^2^ = 0.805 for putamen), and substitution of predicted volumes into the HD-ISS calculator resulted in identical stage classifications compared to the original volumetric data in all test cohort participants. These findings support the clinical utility of the FCR as a feasible alternative to complex volumetric methods for HD-ISS staging.

The current implementation of the HD-ISS depends on the availability of high-quality structural MRI and accurate volumetric quantification of striatal regions. Such data are typically derived from advanced software tools like FreeSurfer, which require standardized acquisition protocols, preprocessing pipelines, and expert oversight. This limits the applicability of the HD-ISS, particularly in non-specialist or resource-limited settings.

In contrast, linear 1D measurements such as FCR and BCR can be obtained rapidly and with minimal training during routine clinical imaging, as confirmed by our interrater reliability analysis and time assessments. While both measures have historically served as surrogate markers for striatal atrophy, our results indicate that the FCR offers stronger predictive value than the BCR for estimating caudate and putamen volumes. This aligns with earlier studies demonstrating that the FCR is sensitive to early frontostriatal changes in HD [3, 4].

Our intention is not to replace volumetric techniques where available, but to propose a low-cost, workflow-compatible proxy that facilitates HD-ISS–aligned staging in routine care. In many clinical settings, automated volumetry is not available at scale due to limitations in infrastructure, turnaround time, or licensing and IT resources. A standardized 1D measurement that captures striatal atrophy and correlates with volumetric surrogates can therefore serve as a practical alternative—supporting triage, longitudinal follow-up, and recruitment, particularly outside centers with extensive MRI expertise.

Possible shortcomings of our study include the small patient group and single centre evaluation limiting generalizability. However, the internal validation with a separate test set and consistent findings across raters lend robustness to the conclusions. In conclusion, the present study indicates that the FCR provides a reliable, time-efficient, and accessible method for estimating striatal atrophy in HD. By enabling HD-ISS staging without the need for complex segmentation tools, it may help expand the reach of standardized disease classification and facilitate clinical research and care in broader clinical contexts.

## Data Availability

Data are available from the corresponding author upon reasonable request.
