# Correction: Simple diameters, accurate stages:a practical approach to HD-ISS staging

**DOI:** 10.1007/s00415-026-13707-2

**Published:** 2026-04-17

**Authors:** Beatrice Heim, Florian Krismer, Greta Hemicker, Clancy Cerejo, Katarina Schwarzova, Samuel Labrecque, Federico Carbone, Marina Peball, Christoph Scherfler, Philipp Mahlknecht, Astrid E. Grams, Elke R. Gizewski, Atbin Djamshidian, Klaus Seppi

**Affiliations:** 1 Department of Neurology, Medical University of Innsbruck, Anichstrasse 35, 6020 Innsbruck, Austria; 2 Department of Radiology, Medical University of Innsbruck, Innsbruck, Austria; 3 Neuroimaging Research Core Facility, Medical University of Innsbruck, Innsbruck, Austria

**Correction: Journal of Neurology** 10.1007/s00415-025-13409-1

In the original version of this article, the Table 1 was incomplete and have now been corrected in the original publication. The old incorrect version are displayed below.

**Table 1:** Demographic characteristics of all participants by clinical disease stage

	preHD	Clinical stage 1	Clinical stage ≥ 2	p-value
Number (n)	15	20	20	< 0.001
Male/Female	4/11	13/7	10/10	0.087
Age (years) (± SD)	37.20 ± 7.36	46.35 ± 12.01	50.00 ± 9.31	< 0.001
UHDRS-TMS (± SD)	1.20 ± 1.70	17.50 ± 7.46	64.56 ± 13.08	< 0.001
UHDRS-TFC (± SD)	13.00 ± 0.00	12.55 ± 0.83	5.56 ± 0.91	< 0.001
CAG repeats (± SD)	43.93 ± 1.49	46.15 ± 4.23	47.10 ± 2.92	0.077
CAP Score (± SD)	515.13 ± 94.86	708.45 ± 71.32	830.25 ± 117.27	< 0.001
PIN_HD_ (± SD)	0.08 ± 0.74	2.41 ± 1.14	4.53 ± 1.74	< 0.001
SDMT (± SD)	49.14 ± 14.15	36.82 ± 16.02	21.14 ± 8.85	< 0.001
Educational years (± SD)	14.01 ± 2.51	13.22 ± 2.80	11.69 ± 2.05	< 0.001

Staging: clinical stage 1: TMS > 5, TFC 13–11; clinical stage ≥ 2: TMS > 5, TFC < 11; preHD, premanifest HD mutation carriers (i.e. TMS < 5)

*n* Number of individuals, *CAG* Cytosine-Adenine-Guanine trinucleotide repeat length in the HTT gene, *UHDRS-TMS* Unified Huntington’s Disease Rating Scale Total Motor Score, *UHDRS-TFC* Unified Huntington’s Disease Rating Scale Total Functional Capacity, *CAP Score* (± SD) CAG-Age Product Score, *PIN*_*HD*_ (± SD) Prognostic Index Normalized for Huntington's Disease, *SDMT* (± SD) Symbol Digit Modalities Test

And also in the Result section, under the subdivision of “Training and test set composition” in the first paragraph the first sentence was incorrectly written as “Of the 55 HD mutation carriers included in the study, 44 were assigned to the training set and 11 to the independent test set. The training cohort comprised mainly manifest patients (n = 40; stage 1: n = 22; stage ≥ 2: n = 18), ensuring a sufficiently large dataset for stable model development.” But should have been “Of the 40 HD mutation carriers included in the study, 40 were assigned to the training set and 4 to the independent test set. The training cohort comprised mainly manifest patients (n = 20; stage 1: n = 22; stage ≥ 2: n = 20), ensuring a sufficiently large dataset for stable model development. In the Discussion, typographical errors affected the reported R^2^ values for the linear regression analyses between FCRand caudate and putamen volumes; the correct R^2^ values are those reported in the Results.

The original article has been corrected.

